# Desmocollin-2 affects the adhesive strength and cytoskeletal arrangement in esophageal squamous cell carcinoma cells

**DOI:** 10.3892/mmr.2014.2485

**Published:** 2014-08-12

**Authors:** WANG-KAI FANG, LIAN-DI LIAO, FA-MIN ZENG, PI-XIAN ZHANG, JIAN-YI WU, JIAN SHEN, LI-YAN XU, EN-MIN LI

**Affiliations:** 1 Department of Biochemistry and Molecular Biology, Shantou University Medical College, Shantou, Guangdong 515041, P.R. China; 2 Key Laboratory of Molecular Biology for High Cancer Incidence Coastal Chaoshan Area, Shantou University Medical College, Shantou, Guangdong 515041, P.R. China; 3 Institute of Oncologic Pathology, Shantou University Medical College, Shantou, Guangdong 515041, P.R. China

**Keywords:** desmocollin-2, esophageal squamous cell carcinoma, cell-cell adhesion, cytoskeleton rearrangement

## Abstract

Desmocollin-2 (DSC2), a transmembrane glycoprotein belonging to the desmosomal cadherin family, has been found to be differentially expressed in several types of cancer and to be involved in tumor progression. The tumor metastasis suppressing property of DSC2 in esophageal squamous cell carcinoma (ESCC) has been described, however, its contribution to cell cohesion in ESCC remains to be elucidated. In the present study, using RNA interference (RNAi), the expression of DSC2 was silenced in SHEEC and KYSE510 cells. Hanging drop and fragmentation assays were performed to investigate the role of DSC2 in cell-cell adhesion. Western blot analysis and confocal microscopy were used to analyze the expression and localization of cell adhesion molecules and cytoskeletal arrangement. The results demonstrated that DSC2 knock down by RNAi caused defects in cell-cell adhesion and a concomitant reduction in desmosomal protein expression and adherens junction molecule distribution. A decrease in the expression of DSC2 caused an increase in free γ-catenin levels, thus promoting its recruitment to the adherens junction complex. In addition, the RNAi-mediated inhibition of DSC2 led to keratin intermediate filament retraction and filamentous-actin cytoskeleton rearrangement. Taken together, these data support our previous findings and the proposal that DSC2 may be involved in the regulation of the invasive behavior of cells by a mechanism that controls cell-cell attachment and cytoskeleton rearrangement.

## Introduction

Adhering junctions, including adherens junctions and desmosomes, are essential for cell unity in sheets of epithelial cells (1). Generally, adhering junctions are comprised of a transmembrane cadherin component, which is involved in homophilic or heterophilic interactions in a subclass-specific manner on the extracellular side and a variety of cytoplasmic adapter proteins that, in turn, link cytoskeletal structures to sites of cell-cell contact (2). The issue of how adhesive dysfunction contributes to cancer biology has become an active area of investigation in cancer research (3). One such protein is desmocollin-2 (DSC2), a transmembrane cadherin of the desmosomal cell-cell adhesion structure. DSC2 is involved in the interaction of plaque proteins and intermediate filaments mediating cell-cell adhesion, and may also contribute to epidermal cell positioning (4).

The desmosomal cadherins family members mediate a variety of biological processes, including cell growth, invasiveness, adhesion and apoptosis (5–8). With respect to tumorigenesis, DSC2 has been demonstrated to be involved in the development of several types of tumor (9–14). A reduction in the expression of DSC2 has been reported in numerous types of human carcinoma, including colorectal, pancreatic, gastric, lung and urothelial cancer (9–14). It has been suggested that a reduction in the expression of DSC2 may act as an independent prognostic biomarker for reduced survival rate in patients (13,14). A previous study of RNA interference (RNAi) in transformed colonic epithelial cells revealed the promotion of tumor cell proliferation *in vitro* and growth *in vivo* following knock down of DSC2 (7).

In human esophageal squamous cell carcinoma (ESCC), the expression of DSC2 has only recently been described (14). Our previous study demonstrated that the expression of DSC2 in ESCC gradually decreases between regions exhibiting esophageal hyperplasia to regions of dysplasia and carcinoma *in situ* (14). The depletion of DSC2 is highly associated with poor tumor differentiation, regional lymph node metastasis and a poor prognosis. Therefore, DSC2 may act as a new molecular marker in predicting the prognosis of patients with ESCC. In addition, our previous study also revealed that DSC2 has a causative effect in esophageal cellular invasion and metastasis (6). The loss of DSC2 initiates tumor cell metastasis by activating the β-catenin pathway and eventually inducing an epithelial-mesenchymal transition-like process (6). However, the contribution of DSC2 to overall cell cohesion remains to be elucidated.

To investigate the possible role of DSC2 in cell-cell adhesion, the present *in vitro* study was performed based on the RNAi strategy in two ESCC cell lines, SHEEC and KYSE510. The results supported our previous findings and the proposal that DSC2 may be involved in the regulation of cell invasion by a mechanism that controls cell-cell attachment and cytoskeleton rearrangement. Altered DSC2 protein levels and localization may, therefore, have several unexpected effects in ESCC.

## Materials and methods

### Cell culture and transfection

The human esophageal squamous carcinoma cell lines SHEEC and KYSE510 (Chinese Academy of Sciences, Shanghai, China) were cultured in Dulbecco’s modified Eagle’s medium (Invitrogen Life Technologies, Carlsbad, CA, USA) supplemented with 10% fetal calf serum (Invitrogen Life Technologies). For siRNA transfection, ~5×10^4^ cells/well were inoculated into 6-well plates, cultured for 24 h and then transfected with the relevant siRNA (50 nM) using a Lipofectamine 2000 transfection reagent (Invitrogen Life Technologies). The siRNA were synthesized by Shanghai GenePharma Co., Ltd. (Shanghai, China) and contained two siRNAs against human DSC2 (siDSC2-1 5′-CUGGAGAUGACAAAGUGUA-3′ and siDSC2-2 5′-CUUUACAGCUGCAAAUCUA-3′). A control siRNA oligonucleotide, not matching any known human coding cDNA, was used as a control.

### RNA extraction and reverse transcription quantitative polymerase chain reaction (RT-qPCR) analysis

Total RNA was extracted using TRIzol reagent (Invitrogen Life Technologies) according to the manufacturer’s instructions. Reverse transcription was performed using a total volume of 20 μl with 1 μg total RNA using a Reverse Transcription system (Promega Corporation, Madison, WI, USA). RT-qPCR was performed on the Rotor-Gene 6000 system (Corbett Life Science, Sydney, Australia). SYBR^®^ Premix Ex Taq™ (Takara Bio, Inc., Shiga, Japan) was used according to the manufacturer’s instructions. The DSC2 PCR primers were designed based on the human DSC2 mRNA sequence (GenBank accession no. NM_024422). The following sequences were used: forward 5′-CCCAAGCTTGAAAAGCCCCTTGGATGAGA-3′ and reverse 5′-CGCGGATCCCCACTGGCTTTCAGAGACTT-3′. As an internal control, a fragment of human β-actin was amplified using the following primers: β-actin, forward 5′-CAACTGGGACGACATGGAGAAA-3′ and reverse 5′-GATAGCAACGTACATGGCTGGG-3′. The PCR conditions were an initial denaturation step of 10 sec at 95°C, followed by 40 cycles consisting of 5 sec at 95°C, 20 sec at 60°C and 15 sec at 72°C.

### Western blot analysis

Total cell lysates were prepared in radioimmunoprecipitation assay buffer, separated by SDS-PAGE and transferred onto polyvinylidene difluoride membranes (Millipore, Billerica, MA, USA). The membranes were incubated in 10 ml of blocking buffer [Tris-buffered saline containing 0.1% (v/v) Tween-20 and 5% (w/v) non-fat dried milk powder (Sangon Biotech, Shanghai, China)] for 1 h at room temperature and then incubated with the indicated antibody. Finally, immunoreactive bands were revealed using a luminol reagent (Santa Cruz Biotechnology, Inc., Santa Cruz, CA, USA). Images were captured and quantitative analyses were performed using FluorChem™ IS-8900 (Alpha Innotech Co., San Leandro, CA, USA). The following antibodies were used: Mouse monoclonal anti-DSC2 (7G6; Invitrogen Life Technologies), mouse monoclonal anti-DSG2 (10G11; Progen Biotechnik GmbH, Heidelberg, Germany), mouse monoclonal anti-PKP2 (PP2/62, PP2/86, PP2/150; Progen Biotechnik GmbH) and mouse monoclonal anti-β-actin (Sigma, St. Louis, MO, USA).

### Confocal laser scanning microscopy

Staining was performed, as previously described (6). The cells were fixed in 4% paraformaldehyde solution (Thermo Fisher Scientific Inc., Rockford, IL, USA) for 15 min, following which they were incubated with donkey serum blocking buffer [Tris-buffered saline containing 5% (w/v) normal donkey serum (Jackson ImmunoResearch, Hamburg, Germany)] for 30 min and the primary antibody for 60 min. Subsequently, the cells were incubated with donkey anti-mouse polyclonal immunoglobulin G (IgG) and/or donkey anti-rabbit polyclonal IgG (DyLight 488 and 594, respectively; Jackson ImmunoResearch) for 60 min at room temperature. The following antibodies were used: Mouse monoclonal anti-DSC2 (7G6; Invitrogen Life Technologies), rabbit polyclonal anti-E-cadherin (Santa Cruz Biotechnology, Inc.), mouse monoclonal anti-γ-catenin (Santa Cruz Biotechnology, Inc.), mouse monoclonal anti-DSG2 (10G11; Progen Biotechnik GmbH), mouse monoclonal anti-PKP2 (PP2/62, PP2/86, PP2/150, Progen Biotechnik GmbH), mouse monoclonal anti-pan-cytokeratin (Santa Cruz Biotechnology, Inc.) and Acti-stain™ 555 Fluorescent Phalloidin (Cytoskeleton Inc., Denver, CO, USA).

### Hanging drop assay

Hanging drop cultures of aggregated cells were generated from 1×10^3^ cells. The cells were left overnight to enable aggregation on the underside of a culture dish, as previously described (15). Following this, pipetting was performed 30 times on the resulting cell clusters through a 200 μl Gilson pipette (Gilson Scientific Ltd., Luton, UK). The degree of dissociation was then determined by counting the number of particles present following trituration.

### Dispase-based dissociation assay

The cell cultures were seeded in triplicate into 6-well plates. Subsequently, 24 h after reaching confluency, the cultures were washed twice in phosphate-buffered saline (PBS) and incubated in 1 ml dispase (2.4 U/ml; Sigma) for 30 min, as previously described (15). The monolayers liberated during this process were carefully washed two times in PBS and then transferred into 15 ml conical tubes. Additional PBS was added to reach a final volume of 2 ml. The tubes were secured to a rocker and subjected to 50 inversion cycles. The fragments were then counted using an inverted microscope (Olympus).

### Statistical analysis

All data are expressed as the mean ± standard deviation and were analyzed using SPSS statistical software (SPSS 13.0; SPSS, Inc., Chicago, IL, USA). Comparisons between data sets were performed using the χ^2^ test and a two-tailed independent sample t-test when appropriate. P<0.05 was considered to indicate a statistically significant difference.

## Results

### Expression of DSC2 in ESCC cell lines

**Figure 1:**
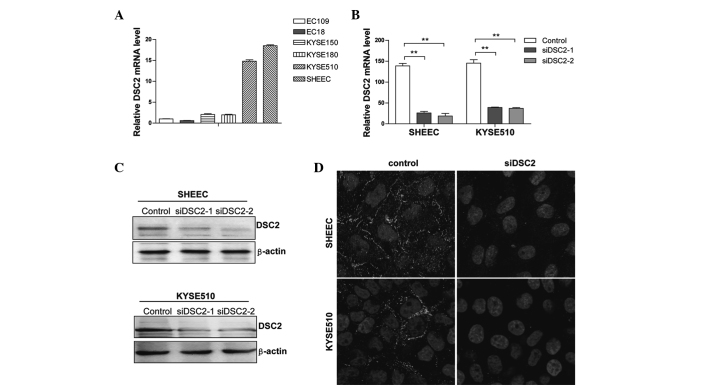
DSC2 silencing in ESCC cells by siRNAs. (A) RT-qPCR analysis of the expression of DSC2 in ESCC cell lines. The expression levels of DSC2 were normalized to that of β-actin. (B) RT-qPCR analysis of DSC2 silencing by siRNAs. Cells were transfected with DSC2 siRNA or control siRNA. (C) DSC2 silencing in SHEEC and KYSE510 cells was evaluated using western blot analysis. β-actin served as a loading control. (D) Immunofluorescence analysis of DSC2 silencing by siRNAs (magnification, ×400). DSC2, desmocollin-2; ESCC, esophageal squamous cell carcinoma; RT-qPCR, reverse transcription quantitative polymerase chain reaction.

Initially, the mRNA expression levels of DSC2 were examined in several ESCC cell lines by RT-qPCR. As shown in Fig. 1A, DSC2 mRNA expression was detected in all the cell lines evaluated, with SHEEC and KYSE510 cells containing the highest levels. Therefore, these cell lines were selected for use as the model in the subsequent functional studies.

### DSC2 knockdown by transient siRNA transfection in ESCC cells

Two double-stranded siRNAs (siRNA-1 and 2) targeting the human DSC2 gene were synthesized. These sequences were specific to DSC2 mRNA and matched no other genes in the NCBI nucleotide database, in particular, they matched no other desmosomal cadherins family members, in BLAST searching. RT-qPCR and western blot analysis revealed that the DSC2 mRNA and protein expression decreased markedly in the treated cells compared with the control cells (Fig. 1B and C). Transfection with DSC2 siRNAs resulted in a 70–80% reduction in the expression of DSC2 in the SHEEC cells (Fig. 1C, upper panel) and a 60–70% reduction in expression in the KYSE510 cells (Fig. 1C, lower panel), when compared with those transfected with the scramble sequence. In addition, confocal analysis of the cells labeled for DSC2 revealed a significant reduction in the membrane localization of DSC2 in the cells treated with siRNA (Fig. 1D).

### DSC2 silencing in ESCC cells compromises cell-cell adhesion

**Figure 2:**
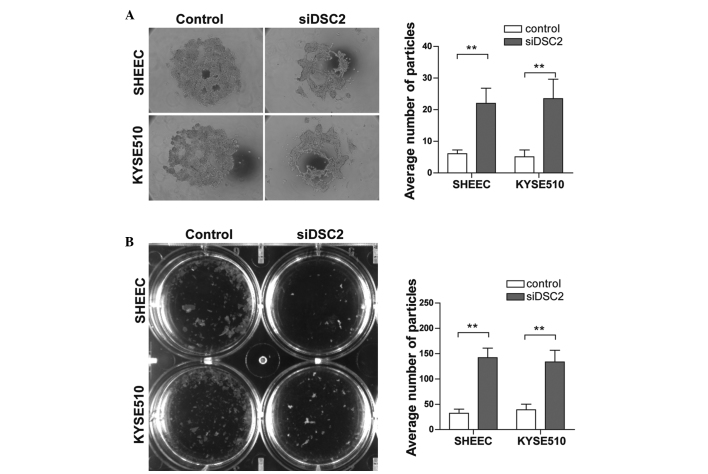
Alterations of cell-cell adhesion exerted by RNA interference-mediated knock down of DSC2. (A) Hanging drop assay. Cells were seeded into hanging drop cultures and allowed to aggregate for 24 h. Following trituration by passing the cell cluster 30 times through a 200 μl pipette tip, the degree of dissociation of the cell cluster was visualized using microscopy and quantified by manual counting using a dissecting microscope. (B) Dispase-based dissociation assay. Cell monolayers were separated from culture dishes via incubation with dispase. Monolayers were transferred to 15 ml conical tubes containing 2 ml phosphate-buffered saline. Following 50 inversions, the degree of fragmentation of the monolayer was examined. The dissociation assay was quantified by counting the number of total particles. ^**^P<0.001, compared with the control group. DSC2, desmocollin-2; siDSC2, DSC2-specific siRNA.

To assess the cell-cell adhesion capacity of ESCC cells following the suppression of DSC2, a hanging drop assay was performed. After 24 h aggregation, in a hanging drop on the underside of a culture dish, the cells were subjected to trituration through a 200 μl Gilson pipette tip to disrupt intercellular adhesion. The degree of dissociation was then calculated by counting the particles that dissociated from the original cluster. The results indicated a significant increase in the number of particles and reduction in particle size in the DSC2-depleted SHEEC and KYSE510 cells (Fig. 2A; P<0.01). To further confirm this effect, a Dispase-based dissociation assay was also performed. Confluent monolayers of siRNA-transfected SHEEC and KYSE510 cells were harvested from tissue culture dishes by incubation with dispase. The monolayers were then transferred into 15-ml conical tubes and, following inversion of the tubes 50 times on a rocker, the monolayer fragments were counted. As shown in Fig. 2B, following siRNA transfection for 2 days, the DSC2-knockdown cells were less cohesive and formed smaller aggregates compared with the control cells (P<0.01).

### Effects of DSC2 depletion on desmosomal proteins and adherens junction molecules

**Figure 3:**
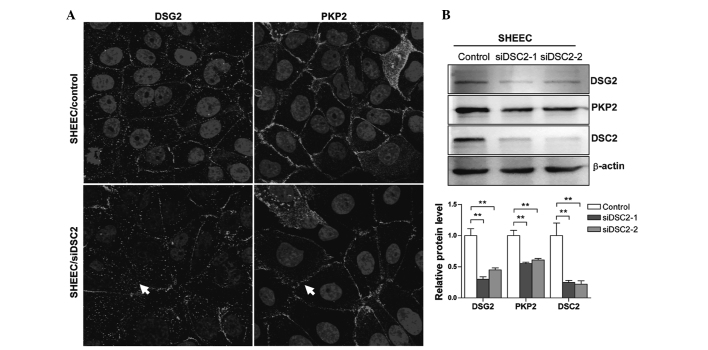
RNA interference-mediated inhibition of DSC2 affects desmosome protein expression and localization. (A) Immunofluorescence analysis of the subcellular localizations of the DSG2 and PKP2 proteins (magnification, ×400). Of note, knocking down the expression of DSC2 caused reduced DSG2 and PKP2 membrane localization (white arrows). (B) Western blot analyses of DSG2 and PKP2 protein expression in the DSC2-specific siRNA or control-transfected SHEEC cells. β-actin served as a loading control. DSC2, desmocollin-2; siDSC2, DSC2-specific siRNA; DSG2, desmoglein-2; PKP2, plakophilin-2.

To assess the effects of RNAi-mediated DSC2 silencing on desmosome junctions in esophageal carcinoma cells, the cells treated with either scrambled or DSC2-specific siRNA were analyzed using fluorescence confocal microscopy to examine the impact on desmosomal junction formation. As shown in Fig. 3A, marked staining for desmoglein-2 (DSG2) and plakophilin-2 (PKP2) was observed in the control cells with localization at the cell membrane. Silencing DSC2 had a marked effect on the membrane localization of these desmosomal proteins and on desmosomal junction formation (Fig. 3A; arrows). To further confirm this effect, the levels of desmosomal protein expression were examined using western blot analysis. The protein expression levels of DSG2 and PKP2 were significantly reduced in the DSC2-specific siRNA-transfected SHEEC cells (Fig. 3B). These results suggested that DSC2 silencing in ESCC cells affects the expression and localization of desmosome proteins and the formation of desmosomal junctions.

**Figure 4:**
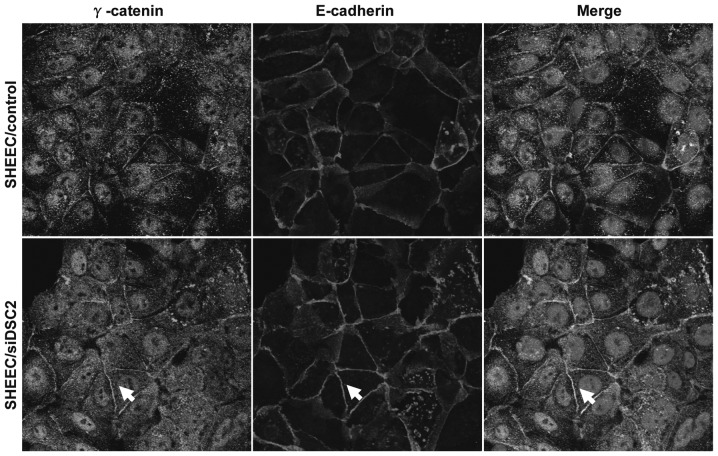
Alterations in the expression of DSC2 reversibly affects the localization of adherens junction molecules. Control siRNA and siDSC2-transfected SHEEC cells were stained using antibodies against γ-catenin and E-cadherin and the representative images are shown. A reduction in DSC2 in the SHEEC cells led to free γ-catenin release and to increased γ-catenin and E-cadherin co-localization (white arrows; magnification, ×400). ^**^P<0.001, compared with the control group. DSC2, desmocollin-2; siDSC2, DSC2-specific siRNA.

Desmosomes and adherens junctions have associated effects in maintaining cellular adhesion and have similar structural compositions. In addition, E-cadherin and DSC2 protein bind directly to an armadillo family member, γ-catenin (16). Therefore, the present study investigated the effects of DSC2 knock down on the localization of γ-catenin and E-cadherin. As shown in Fig. 4, depletion of DSC2 resulted in increased levels of free γ-catenin levels, promoting the recruitment of γ-catenin to the adherens junction complex. This contributes to our previous findings and supports the concept that DSC2 may be involved in the regulation of cell invasive behavior by a mechanism that controls cell-cell attachment, including adherens junctions and desmosomes.

### DSC2 depletion leads to keratin intermediate filament retraction and filamentous (F)-actin cytoskeleton rearrangement

**Figure 5:**
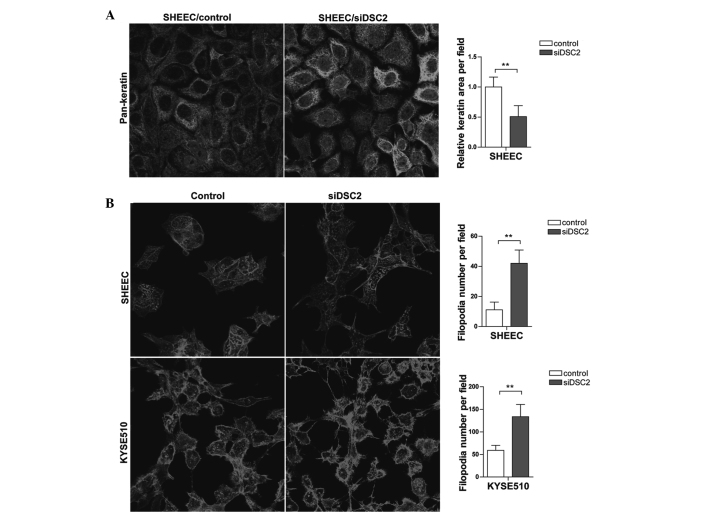
DSC2 depletion leads to keratin intermediate filament retraction and F-actin cytoskeleton rearrangement. (A) Immunofluorescence analysis shows retracted keratin intermediate filaments from plasma membranes in siDSC2-transfected SHEEC cells, whereas the plasma membranes of neighboring cells remained adjacent in the control-transfected SHEEC cells (magnification, ×400). (B) SHEEC and KYSE510 cells were transiently transfected with control siRNA and siDSC2. The transfected cells were then fixed and F-actin organization was analyzed using phalloidin staining (magnification, ×400). DSC2, desmocollin-2; siDSC2, DSC2-specific siRNA.

Tumor metastasis requires the rearrangement of the adhesive contacts of tumor cells to enable cells to migrate relative to neighboring cells It has been suggested that, in this process, actin filament and intermediate filament-based junctions act synergistically to affect cell-cell adhesion (15). Therefore, the present study investigated whether DSC2 depletion affects cytoskeleton rearrangement. Immunofluorescence analysis revealed retracted keratin intermediate filaments from the plasma membranes in DSC2-specific siRNA-transfected SHEEC cells, whereas, in cells transfected with the scramble sequence, the keratin intermediate filaments lining the plasma membranes remained (Fig. 5A). Furthermore, phalloidin-coumarin staining indicated that DSC2 depletion affected the F-actin arrangement (Fig. 5B). Compared with the control group, the cells with reduced DSC2 exhibited more filopodia. These results indicated that DSC2 depletion resulted in cytoskeleton rearrangement, ultimately promoting cell invasive behavior.

## Discussion

The present study is one of the first attempts, to the best of our knowledge, to associate the expression of DSC2 with cell-cell adhesion and cytoskeleton rearrangement in ESCC. Several studies have found that DSC2 proteins are abnormally expressed in various types of cancer and correlate with cell proliferation and invasive behavior (6,7). However, fewer studies have focused on investigating the role of DSC2 in regulating adhesive strength. In the present study, an RNAi strategy was adopted to investigate whether DSC2 has a role in the regulation of cell-cell adhesion in ESCC cell lines. Using various approaches, the present study demonstrated that knock down of DSC2 in these cell lines caused defects in cell-cell adhesion, accompanied by reduced desmosomal junction formation, retraction of keratin intermediate filaments and F-actin cytoskeleton rearrangement.

Our previous studies have demonstrated that DSC2 has a causative effect in cellular invasion and metastasis in laboratory models and in clinical ESCC samples (6,14). DSC2 depletion is highly associated with poor tumor differentiation, regional lymph node metastasis and poor prognosis. In the present study, the results revealed that DSC2 may be involved in the regulation of cell invasive behavior by a mechanism that controls cell-cell attachment, including adherens junctions and desmosomes. As a junctional protein, DSC2 interacts extracellularly with DSG2/3 to mediate cell-cell adhesion (17). It has been suggested that specific desmosomal cadherins have a different contribution in cell-cell adhesion (18). Consistent with this observation, the data presented in the present study suggested that a decreased level of DSC2 in ESCC cells leads to a decrease in the expression of desmosomal proteins and desmosomal junction formation, which was accompanied by a decrease in cell-cell attachment. These results are consistent with data from Mannan *et al* that DSG3 silencing in HaCaT cells causes disruption of desmosome junctions and compromises cell-cell adhesion (19). This leads to the conclusion that knocking down the expression of DSC2 by siRNA destabilizes other desmosomal proteins. Although, it remains unclear whether this represents transcriptional downregulation or increased protein turnover, possibly complexed with DSC2, the reduction of cell adhesion protein suggests that the inhibition of cell-cell adhesion is partly dependent upon the interaction of DSC2 with other desmosomal molecules.

Previous studies have revealed that adherens junctions and desmosomes are dependant on each other for appropriate assembly and maintenance (15,16). In addition to their independent roles in tissue morphogenesis and homeostasis, adherens junctions and desmosomes-based cell-cell adhesive contacts engage in ‘cross-talk’ mechanisms, in which one junction type affects the expression, assembly, turnover and/or function of the other junctions or junction components. It has been suggested that achieving maximal mechanical integrity of the epithelial cell sheet requires proper junctional attachment to intermediate filaments and cortical actin (15). Furthermore, it has been demonstrated that the loss of desmosomal components leads to defects in the maturation of adherens junctions and the associated cortical actin cytoskeleton (20). In comparison, the present study demonstrated that DSC2 suppresses tumor cell-cell adhesion by opposing the localization of adherens junction molecules and keratin intermediate filament retraction and F-actin cytoskeleton rearrangement. DSC2 depletion from the desmosome structure may compromise adhesive strength in several ways. DSC2 may destabilize the desmosomal cadherin clusters formed and affect the proper disposition of desmosomal cadherin extracellular domains, as discussed previously. Another possibility is that DSC2 anchors the armadillo repeat-containing proteins γ-catenin and PKP2 that, in turn, recruit the keratin intermediate filament cytoskeleton to sites of cell-cell contact. Uncoupling of these connections results in increased levels of free γ-catenin and PKP2. It has been suggested that PKP2 may functionally link the Ras homolog (Rho)A and protein kinase C-dependent pathways to drive actin reorganization and to regulate the assembly of desmosomes (21,22). PKP2 may provide a structural link between junctional components and the actin and the intermediate filament cytoskeletons to coordinate their activities during assembly of junctions. In addition, γ-catenin is required for effective anchorage of intermediate filaments to desmosomes (23). A study by Todorović *et al* reported that γ-catenin regulates cell motility through Rho-and fibronectin-dependent Src signaling (24). Therefore, in esophageal carcinoma cells, DSC2 may affect cytoskeleton rearrangement via alternative signaling pathways, which requires extensive investigation to determine.

Taken together, the present study suggests that DSC2 is important in the regulation of cell-cell adhesion dependent motility. Depletion of the transmembrane desmosomal cadherin components is likely to occur by destabilizing other desmosomal proteins. Uncoupling of these connections resulted in increased levels of free desmosomal plaque proteins. This in turn affected cytoskeleton arrangement, possibly through alternative signaling pathways, which ultimately led to uncontrolled migration of tumor cells lacking DSC2.

## References

[1] Green KJ, Getsios S, Troyanovsky S, Godsel LM (2010). Intercellular junction assembly, dynamics, and homeostasis. Cold Spring Harb Perspect Biol.

[2] Angst BD, Marcozzi C, Magee AI (2001). The cadherin superfamily: diversity in form and function. J Cell Sci.

[3] Jeanes A, Gottardi CJ, Yap AS (2008). Cadherins and cancer: how does cadherin dysfunction promote tumor progression?. Oncogene.

[4] Nuber UA, Schäfer S, Schmidt A, Koch PJ, Franke WW (1995). The widespread human desmocollin Dsc2 and tissue-specific patterns of synthesis of various desmocollin subtypes. Eur J Cell Biol.

[5] Chen YJ, Chang JT, Lee L, Wang HM, Liao CT, Chiu CC, Chen PJ, Cheng AJ (2007). DSG3 is overexpressed in head neck cancer and is a potential molecular target for inhibition of oncogenesis. Oncogene.

[6] Fang WK, Liao LD, Li LY, Xie YM, Xu XE, Zhao WJ, Wu JY, Zhu MX, Wu ZY, Du ZP, Wu BL, Xie D, Guo MZ, Xu LY, Li EM (2013). Down-regulated desmocollin-2 promotes cell aggressiveness through redistributing adherens junctions and activating beta-catenin signalling in oesophageal squamous cell carcinoma. J Pathol.

[7] Kolegraff K, Nava P, Helms MN, Parkos CA, Nusrat A (2011). Loss of desmocollin-2 confers a tumorigenic phenotype to colonic epithelial cells through activation of Akt/{beta}-catenin signaling. Mol Biol Cell.

[8] Dusek RL, Getsios S, Chen F, Park JK, Amargo EV, Cryns VL, Green KJ (2006). The differentiation-dependent desmosomal cadherin desmoglein 1 is a novel caspase-3 target that regulates apoptosis in keratinocytes. J Biol Chem.

[9] Khan K, Hardy R, Haq A, Ogunbiyi O, Morton D, Chidgey M (2006). Desmocollin switching in colorectal cancer. Br J Cancer.

[10] Anami K, Oue N, Noguchi T, Sakamoto N, Sentani K, Hayashi T, Hinoi T, Okajima M, Graff JM, Yasui W (2010). Search for transmembrane protein in gastric cancer by the *Escherichia coli* ampicillin secretion trap: expression of DSC2 in gastric cancer with intestinal phenotype. J Pathol.

[11] Cui T, Chen Y, Yang L, Mireskandari M, Knösel T, Zhang Q, Kohler LH, Kunze A, Presselt N, Petersen I (2012). Diagnostic and prognostic impact of desmocollins in human lung cancer. J Clin Pathol.

[12] Hayashi T, Sentani K, Oue N, Anami K, Sakamoto N, Ohara S, Teishima J, Noguchi T, Nakayama H, Taniyama K, Matsubara A, Yasui W (2011). Desmocollin 2 is a new immunohistochemical marker indicative of squamous differentiation in urothelial carcinoma. Histopathology.

[13] Hamidov Z, Altendorf-Hofmann A, Chen Y, Settmacher U, Petersen I, Knösel T (2011). Reduced expression of desmocollin 2 is an independent prognostic biomarker for shorter patients survival in pancreatic ductal adenocarcinoma. J Clin Pathol.

[14] Fang WK, Gu W, Li EM, Wu ZY, Shen ZY, Shen JH, Wu JY, Pan F, Lv Z, Xu XE, Huang Q, Xu LY (2010). Reduced membranous and ectopic cytoplasmic expression of DSC2 in esophageal squamous cell carcinoma: an independent prognostic factor. Hum Pathol.

[15] Huen AC, Park JK, Godsel LM, Chen X, Bannon LJ, Amargo EV, Hudson TY, Mongiu AK, Leigh IM, Kelsell DP, Gumbiner BM, Green KJ (2002). Intermediate filament-membrane attachments function synergistically with actin-dependent contacts to regulate intercellular adhesive strength. J Cell Biol.

[16] Lewis JE, Wahl JK, Sass KM, Jensen PJ, Johnson KR, Wheelock MJ (1997). Cross-talk between adherens junctions and desmosomes depends on plakoglobin. J Cell Biol.

[17] Garrod D, Chidgey M (2008). Desmosome structure, composition and function. Biochim Biophys Acta.

[18] Hartlieb E, Kempf B, Partilla M, Vigh B, Spindler V, Waschke J (2013). Desmoglein 2 is less important than desmoglein 3 for keratinocyte cohesion. PLoS One.

[19] Mannan T, Jing S, Foroushania SH, Fortune F, Wan H (2011). RNAi-mediated inhibition of the desmosomal cadherin (desmoglein 3) impairs epithelial cell proliferation. Cell Prolif.

[20] Vasioukhin V, Bowers E, Bauer C, Degenstein L, Fuchs E (2001). Desmoplakin is essential in epidermal sheet formation. Nat Cell Biol.

[21] Bass-Zubek AE, Hobbs RP, Amargo EV, Garcia NJ, Hsieh SN, Chen X, Wahl JK, Denning MF, Green KJ (2008). Plakophilin 2: a critical scaffold for PKC alpha that regulates intercellular junction assembly. J Cell Biol.

[22] Godsel LM, Dubash AD, Bass-Zubek AE, Amargo EV, Klessner JL, Hobbs RP, Chen X, Green KJ (2010). Plakophilin 2 couples actomyosin remodeling to desmosomal plaque assembly via RhoA. Mol Biol Cell.

[23] Acehan D, Petzold C, Gumper I, Sabatini DD, Müller EJ, Cowin P, Stokes DL (2008). Plakoglobin is required for effective intermediate filament anchorage to desmosomes. J Invest Dermatol.

[24] Todorović V, Desai BV, Patterson MJ, Amargo EV, Dubash AD, Yin T, Jones JC, Green KJ (2010). Plakoglobin regulates cell motility through Rho- and fibronectin-dependent Src signaling. J Cell Sci.

